# Supplementary material for: Transcriptional Landscape of Waddlia chondrophila Aberrant Bodies Induced by Iron Starvation
Source: Microorganisms. 2020 Nov 24;8(12):1848. doi: 10.3390/microorganisms8121848 (PMC7760296; doi:10.3390/microorganisms8121848)
Supplement: Supplementary file 1 [file microorganisms-08-01848-s001.zip › Supplementary Material.docx]

**
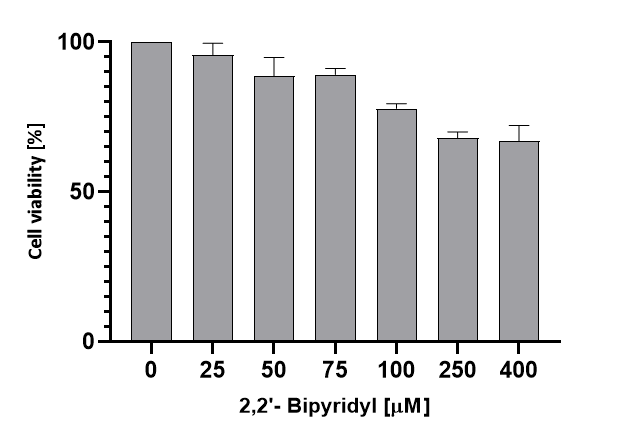
**

**Supplementary Figure 1. Viability of HEp-2 cells in presence of 2,2’-bipyridyl.** Viability of cells grown in presence of different concentrations of BPDL for 24 hours was assessed using Resazurin. Results are shown as percentages, with the untreated control set at 100%, and represent the mean and standard deviation of triplicates measured in one experiment.

**
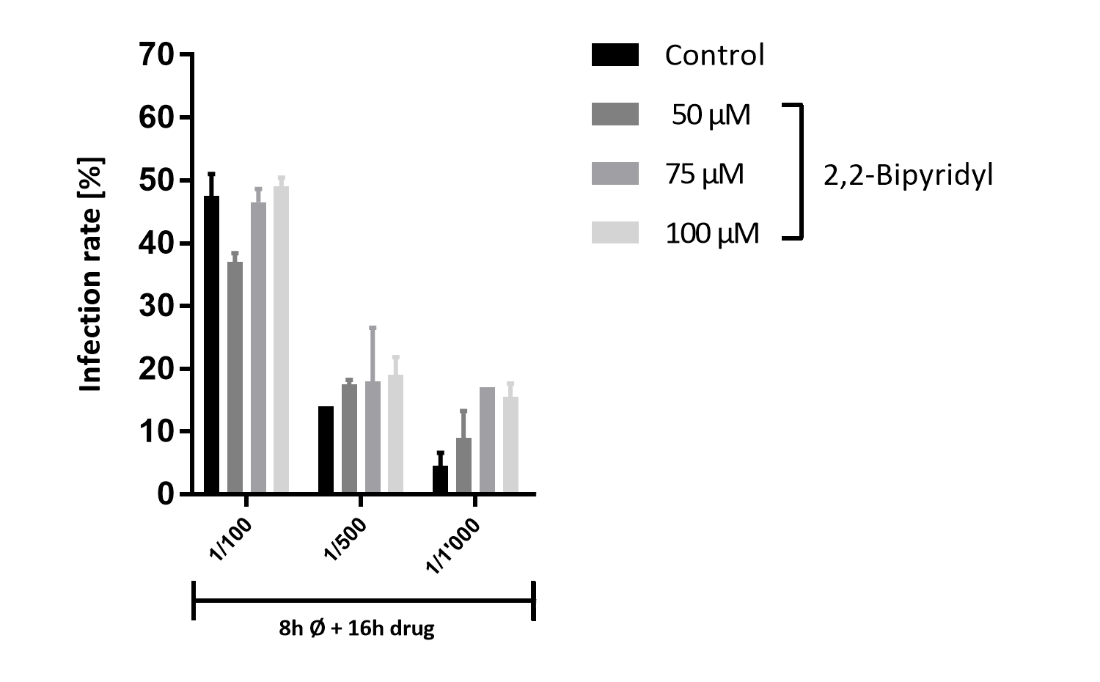
**

**Supplementary Figure 2. Infection rate obtained with different dilutions of the *W. chondrophila* inoculum.** HEp-2 cells were infected with *W. chondrophila* (different dilutions of the co-culture filtrate from *A. castellanii*), treated with different concentrations of BPDL 8 hpi and fixed 24 hpi. Fixed cells were labelled with DAPI and anti-*W. chondrophila* antibodies in order to count the number of cells and inclusions in the field by confocal microscopy. A dilution of the inoculum of 1:100 (corresponding to an MOI of 15) was chosen for further experiments, as it provided an infection rate of 40-50% for all the conditions tested (in particular untreated and treated with 75 μM BPDL, the conditions used for the RNA-seq).

**
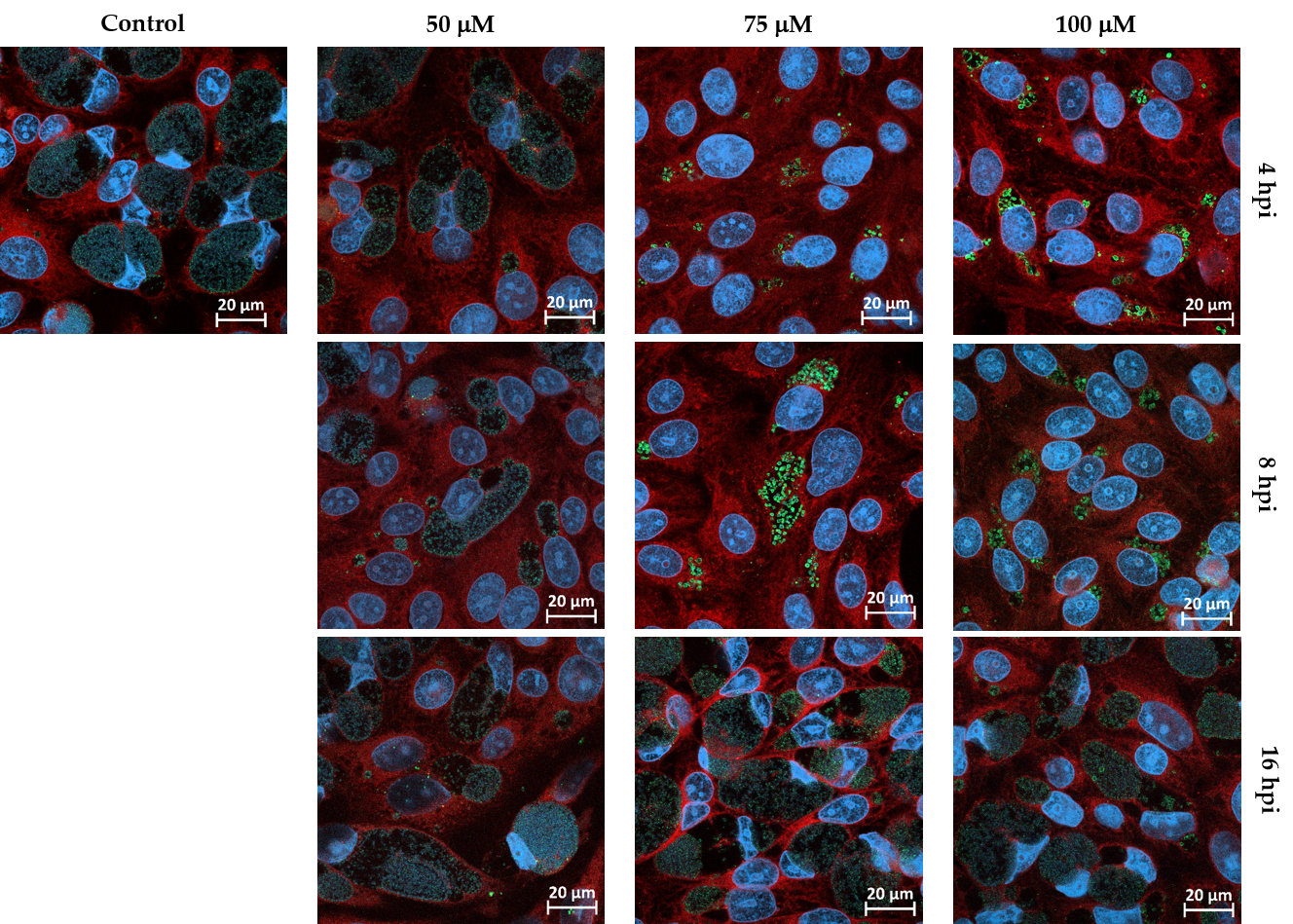
**

**Supplementary Figure 3. Aberrant bodies formation in the presence of BPDL depends on the concentration and time of addition of BPDL.** HEp-2 cells were infected with *W. chondrophila* and treated with 50, 75 or 100 μM BPDL 4, 8 or 16 hpi. Infected cells were fixed 24 hours pi, labelled with Concanavalin A (red), DAPI (blue) and anti-*W. chondrophila* antibodies (green) and observed by confocal microscopy. When added 16 hpi, BPDL did not induce the formation of ABs. Addition of BPDL (75 or 100 μM) at 4 hpi determined the formation of only a few ABs per inclusion. Treatment with 75 μM BPDL 8 hpi induced the formation of numerous ABs, thus it was chosen for the RNA-seq experiment. Images of HEp-2 cells infected with *W. chondrophila* and treated with BPDL at 2 hpi are shown in **Figure 1**.

**
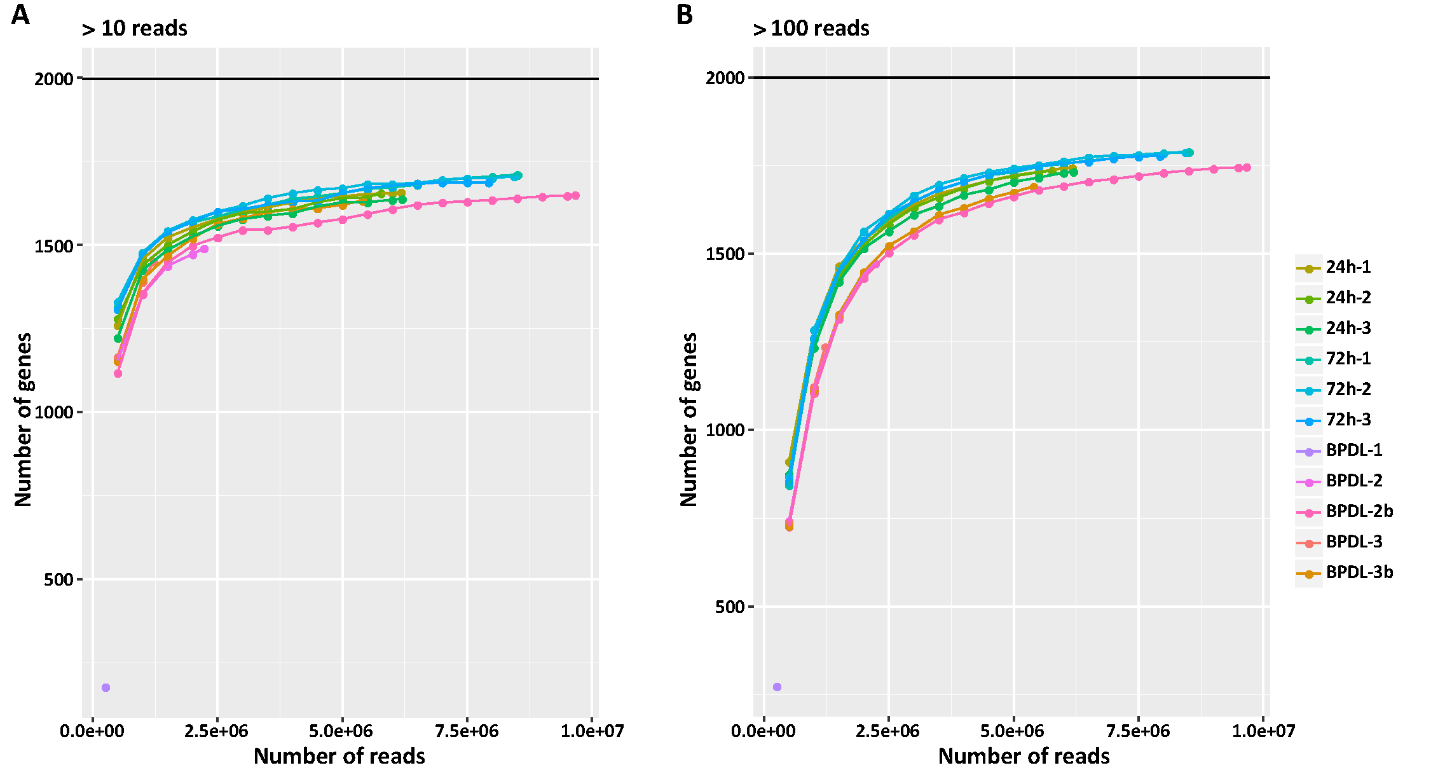
****Supplementary Figure 4: Rarefaction analysis on the RNA sequencing samples.** The graphs indicate the number of genes mapped by at least 10 (left panel) or 100 reads (right panel). Triplicates were used for each condition. Due to the low number of reads (BPDL-2 and BPDL-3), the sequencing and mapping were repeated for two samples (BPDL-2b and BPDL-3b). BPDL-1 sample was removed from the analysis. The absence of reads mapping on some genes indicates that these genes are very lowly expressed under the considered condition, or that the annotation of the genome at these loci is incorrect.

**
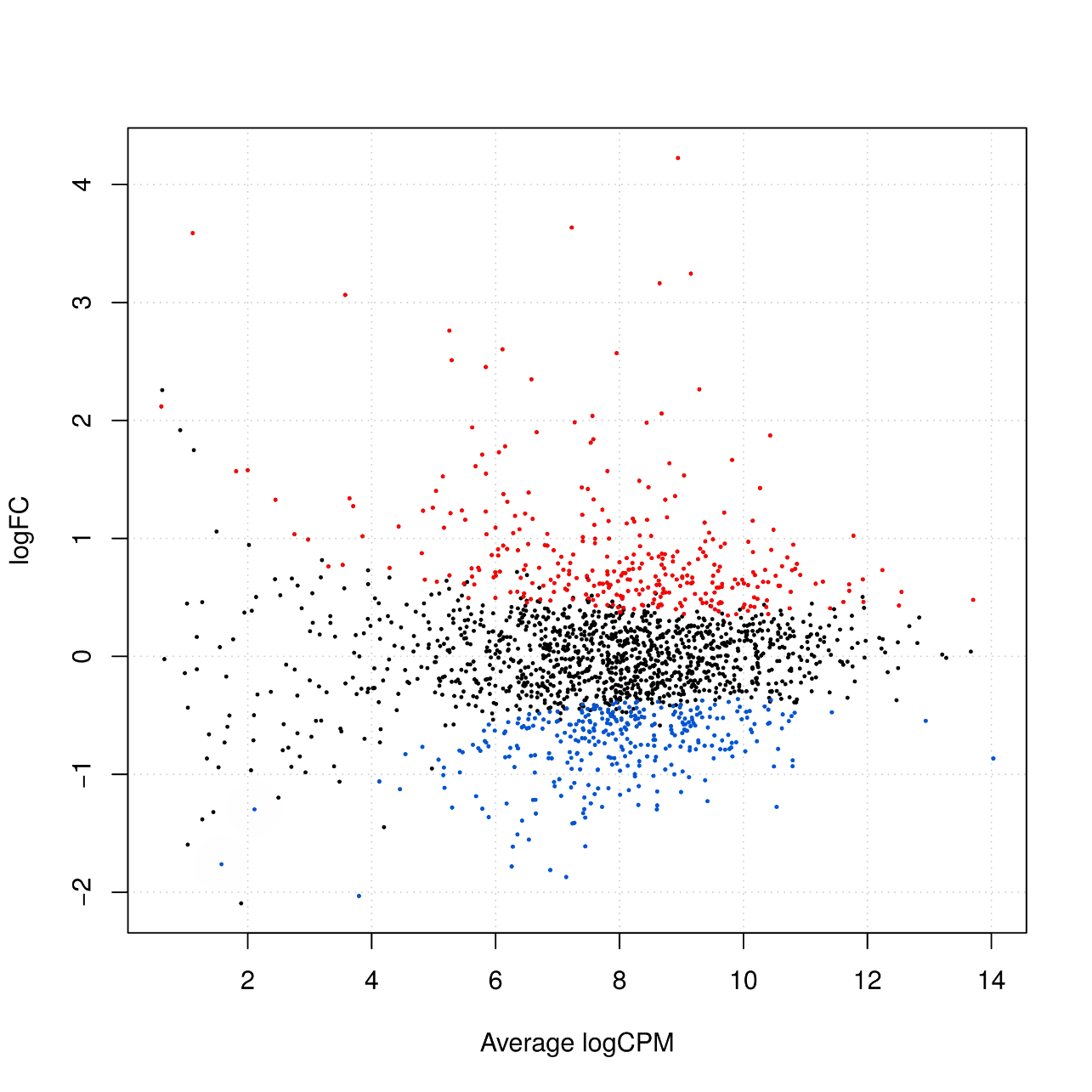
 Supplementary Figure 5: Gene expression in BPDL-treated *W. chondrophila* versus untreated RBs.** The smear plot indicates the relation between the log2 fold change and the average log counts per million. Each point represents the average value for one gene. Significantly up-regulated genes are in red whereas significantly down-regulated genes are in blue (FDR ≤ 0.01).


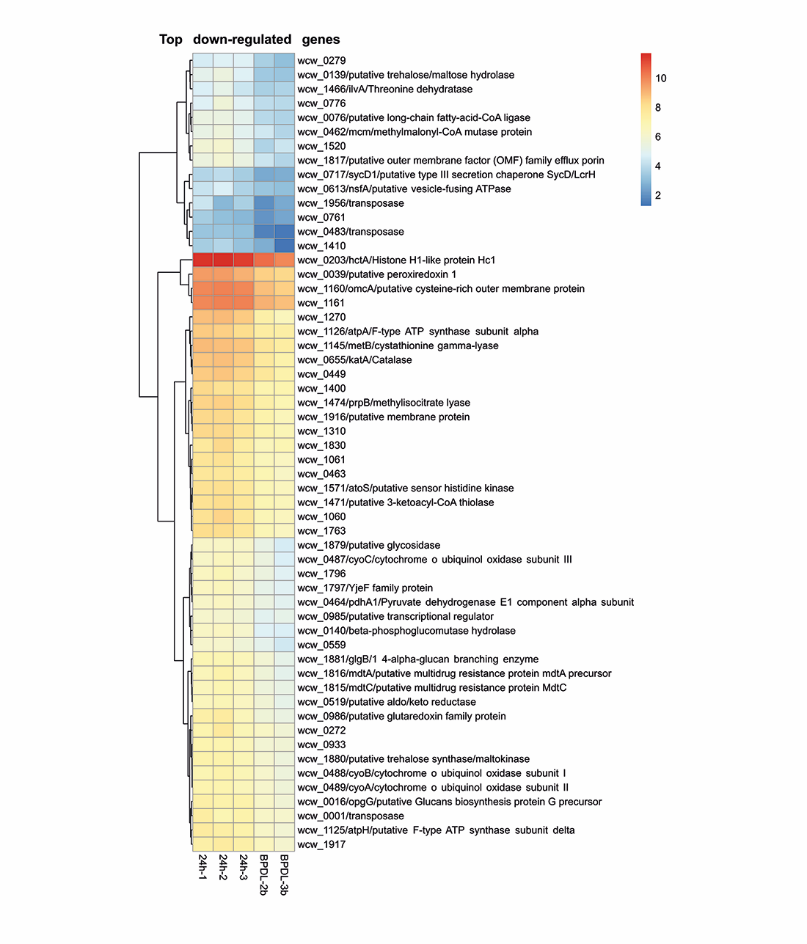
 **Supplementary Figure 6. Normalized expression profile of the top down-regulated genes in response to iron deprivation.** The heatmap displays significantly down-regulated genes exhibiting at least a twofold decreased expression in BPDL-treated samples as compared to the untreated control (log2FC ≤ -1; FDR ≤ 0.01). In total, 56 genes were significantly down-regulated in *W. chondrophila* aberrant bodies. Red indicates higher expression values whereas blue corresponds to lower expression values.


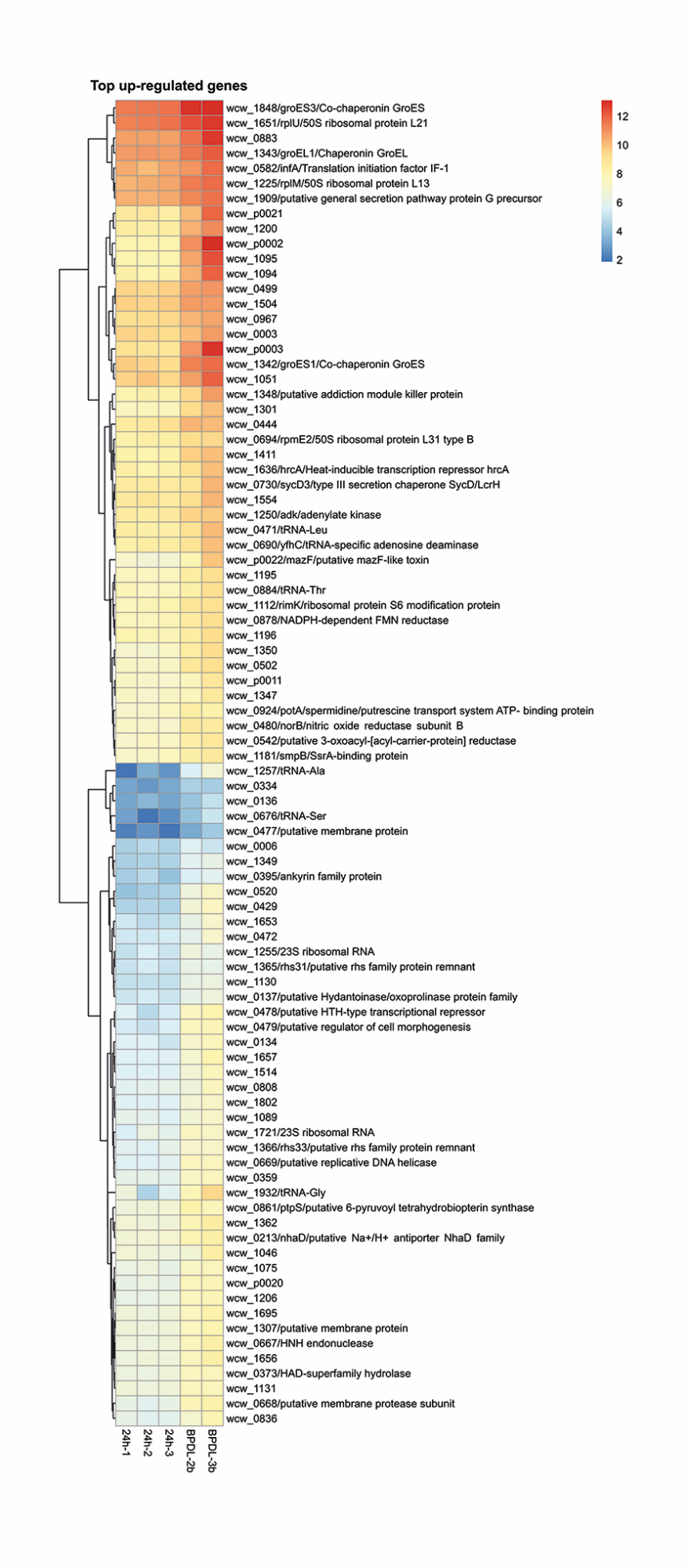


**Supplementary Figure 7. Normalized expression profile of the top up-regulated genes in response to iron deprivation.** The heatmap shows significantly up-regulated genes exhibiting at least a twofold increased expression in BPDL-treated samples compared to the untreated control (log2FC ≥ 1; FDR ≤ 0.01). In total, 88 genes were significantly up-regulated in *W. chondrophila* aberrant bodies. Red colour indicates higher expression values whereas blue corresponds to lower expression values.

**Supplementary Table 1 (.xlsx file).** RNA-seq data. The counts and fold change (BPDL-treated vs untreated control, expressed as log2FC) of all the genes whose transcripts were detected by RNA-seq are reported in Table S1. Up-regulated genes: list of the 332 significantly up-regulated (FDR ≤ 0.01) genes. Genes with a log2FC ≥ 1 are in bold. Genes with a log2FC ≥ 2 are in red. Down-regulated genes: list of the 325 significantly down-regulated (FDR ≤ 0.01) genes. Genes with a log2FC ≥ 1 are in bold. Genes with a log2FC ≥ 2 are in blue.

**Table S2 (.xlsx file).** Gene ontology enrichment analysis of down-regulated genes. Table S2 contains detailed results of the Gene ontology enrichment analysis with raw and corrected p-values, as well as the list of loci annotated with the corresponding GO term. Abbreviations: BP, Biological Process; MF, Molecular Function; CC, Cellular Component; e, enriched term; p, under-represented term.

**Table S3 (.xlsx file).** Gene ontology enrichment analysis of up-regulated genes. Table S3 contains detailed results of the Gene ontology enrichment analysis with raw and corrected p-values, as well as the list of loci annotated with the corresponding GO term. Abbreviations: BP, Biological Process; MF, Molecular Function; CC, Cellular Component; e, enriched term; p, under-represented term.
